# Facharztausbildung in Deutschland und Österreich im Vergleich: Der Blick über die Grenze eröffnet neue Perspektiven: Karrierepfade und Chancen

**DOI:** 10.1007/s00292-025-01504-z

**Published:** 2025-11-13

**Authors:** Steffen Ormanns, Andrea Brunner-Véber, Michael Günther

**Affiliations:** 1 Institut für Allgemeine Pathologie, Medizinische Universität Innsbruck, Müllerstraße 44, 6020 Innsbruck, Österreich; 2 Tirol Kliniken, Innpath Institut für Pathologie, Anichstraße 35, 6020 Innsbruck, Österreich

**Keywords:** Pathologie, Wissenschaft, Berufseinstieg, Facharztprüfung, Pathology, Science, Career entry, Specialist examination

## Abstract

Aufgrund der räumlichen Nähe und der gegenseitigen Anerkennung des Facharztes kann eine Facharztausbildung in Österreich für Berufseinsteiger attraktiv erscheinen.

Für einen Wechsel ist das Wissen um die Unterschiede im Aufbau und den Anforderungen der Facharztausbildung sowie den Möglichkeiten für eine wissenschaftliche Karriere von großer Bedeutung.

Dabei setzt die Facharztausbildung in Österreich eine verpflichtende neunmonatige Basisausbildung voraus und ist durch eine strikte Aufteilung in eine Grundausbildung und eine Schwerpunktausbildung in der anschließenden Facharztausbildung stärker strukturiert. Daneben bietet die Schwerpunktausbildung den Auszubildenden einen größeren Gestaltungsspielraum, da sie hier ihre Ausbildung besser an die individuellen Bedürfnisse und Ziele anpassen können.

Zudem zeigen sich geringe Unterschiede in den wissenschaftlichen Entwicklungsmöglichkeiten, die in die Entscheidung einbezogen werden sollten.

## Abstract (en)

Young professionals may find it appealing to pursue specialist training in Austria due to the country’s geographical proximity and the mutual recognition of medical specialists.

When considering a career change, it is important to be aware of the differences in the structure and requirements of specialty training programs and in scientific career opportunities.

Specialist training in Austria requires the completion of a mandatory nine-month basic training program. It is more structured, with a strict division between basic training and specialized training in the subsequent specialist training program. The specialization training also offers greater flexibility for trainees, allowing them to customize their learning experience and advance their skills in a way that suits their individual needs and goals.

In addition, before making a final decision, one must take into account the disparities in scientific development prospects.

Die räumliche Nähe von Deutschland und Österreich lässt für Studierende und Ärzte, die eine Facharztausbildung im Fach Pathologie anstreben, eine Facharztausbildung in Österreich attraktiv erscheinen. Dies wird auch durch die gegenseitige Anerkennung des Facharzttitels verstärkt.

Daher ist die Kenntnis über Unterschiede in den Ausbildungssystemen zwischen den beiden Ländern von großer Bedeutung, da diese Einfluss auf die Entwicklungsmöglichkeiten am jeweiligen Standort nehmen können.

## Facharztausbildung in Österreich

**Abb. 1:**
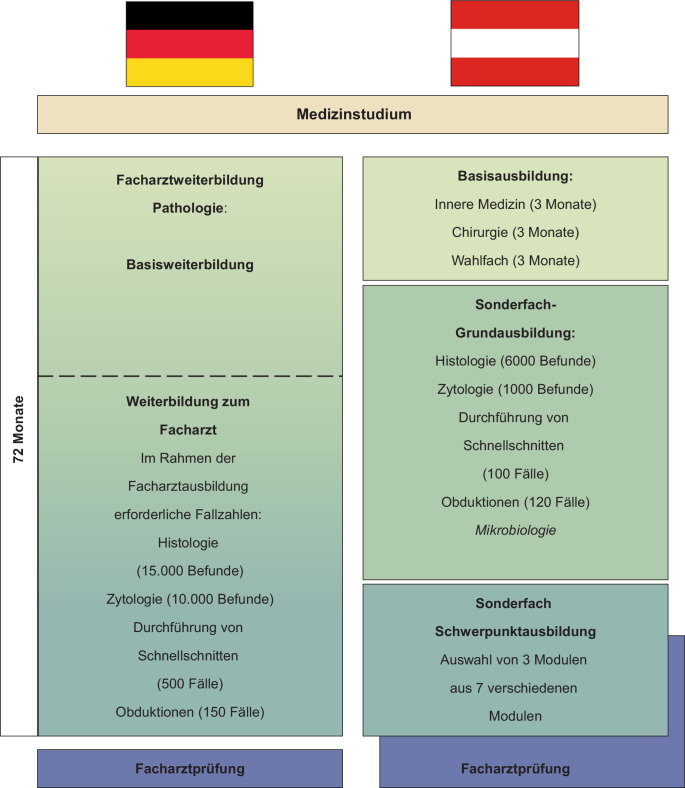
Übersicht über den Aufbau der Facharztausbildung in der Pathologie in Deutschland und Österreich

Die Facharztausbildung in Österreich lässt sich in 3 Abschnitte untergliedern (Abb. 1).

Vor Beginn der eigentlichen Facharztausbildung ist die Absolvierung der neunmonatigen Basisausbildung verpflichtend erforderlich. Diese untergliedert sich in Abschnitte von jeweils 3 Monaten, von denen ein Abschnitt in einer Abteilung der Inneren Medizin, ein Abschnitt in einer chirurgischen Abteilung und ein Abschnitt in einem klinischen Wahlfach absolviert werden muss. Da das Fach Pathologie und Molekularpathologie in Österreich generell nicht als „klinisch“ betrachtet wird, kann in diesem Fach kein Tertial der Basisausbildung abgeleistet werden bzw. wird es nicht als Wahlfach für die Basisausbildung von den Ärztekammern anerkannt.

Die Basisausbildung ist auch bei einem Wechsel nach Österreich während der Facharztausbildung zu durchlaufen. So kann bei einem Wechsel eine bereits in Deutschland begonnene Facharztausbildung erst nach erfolgreichem Abschluss der Basisausbildung fortgesetzt werden. Die Absolvierung von Nacht- oder Wochenenddiensten ist ein verpflichtender Bestandteil der Basisausbildung.

Nach Abschluss der Basisausbildung kann die Facharztausbildung im Fach der klinischen Pathologie und Molekularpathologie begonnen oder fortgesetzt werden. Diese wird zu den sogenannten Sonderfächern gezählt.

Die Facharztausbildung beginnt mit der 36-monatigen Sonderfach-Grundausbildung, in deren Rahmen sollen grundlegende fachliche Fähigkeiten vermittelt werden. Diese bestehen zum einen neben allgemeinen Kompetenzen und dem Erwerb der Kenntnis der Rechtsvorschriften aus der histologischen Untersuchung von 6000 Fällen, von denen zumindest 1000 eine „aufwendige Präparation“ erfordern, was nicht weiter definiert ist. Daneben sollen 1000 zytodiagnostische Untersuchungen, 100 Schnellschnittuntersuchen und 120 Obduktionen durchgeführt werden. Die Ausbildungsordnung sieht auch 100 Befunde mit speziellen Färbe- und Analysetechniken wie immunhistochemischen Färbungen oder molekulargenetischen Untersuchungen vor. Im Unterschied zu einer Facharztausbildung in Deutschland sind in Österreich auch Kompetenzen im Bereich der Mikrobiologie zu erwerben.

Nach Abschluss der Sonderfach-Grundausbildung beginnt die 27-monatige Sonderfach-Schwerpunktausbildung. Diese ist aus 3 jeweils neunmonatigen Modulen aufgebaut, die aus 6 fachspezifischen Modulen sowie dem wissenschaftlichen Modul ausgewählt werden können.

Die verschiedenen fachspezifischen Module (Spezielle Pathologie solider Neoplasien, Spezielle Pathologie nichtneoplastischer Erkrankungen, Hämatopathologie, Molekularpathologie, Klinische Mikrobiologie und Klinische Zytopathologie) weisen entsprechend der jeweiligen fachlichen Ausrichtung starke Unterschiede hinsichtlich der Anforderungen an die Auszubildenden auf. So variieren die Fallzahlen zwischen 2000 Befunden in den Modulen „Spezielle Pathologie solider Neoplasien“ und „Spezielle Pathologie nichtneoplastischer Erkrankungen“ und keinerlei histologischen Befunden im Modul „Klinische Mikrobiologie“.

Von den fachspezifischen Modulen muss das wissenschaftliche Modul abgegrenzt werden. Dieses erfordert die Durchführung mindestens eines Forschungsprojektes und soll den Auszubildenden die Möglichkeit bieten, ihr wissenschaftliches Profil zu stärken. Die Absolvierung des wissenschaftlichen Moduls kann sich naturgemäß an nichtakademischen Zentren unter Umständen als problematisch erweisen.

Da die Facharztprüfung nur einmal jährlich zentral für ganz Österreich abgehalten wird, besteht die Möglichkeit, sich bereits nach 44 Monaten in der Facharztausbildung beginnend mit der Basisausbildung zur Prüfung anzumelden. Dies hat zur Konsequenz, dass man sich bereits einen Monat vor Ablauf der Sonderfach-Grundausbildung zur Prüfung anmelden und diese dann im zweiten bzw. dritten Monat des ersten Abschnitts der Sonderfach-Schwerpunktausbildung absolvieren kann. Nach Bestehen der Prüfung darf der Facharzttitel jedoch erst nach vollständiger Absolvierung der Ausbildungszeit und Erfüllung aller Fallzahlen geführt werden. Die Facharztprüfung besteht aus 28 Fallbeispielen, überwiegend basierend auf verschiedenen Präparaten mit bis zu 6 Unterfragen, die in 4 h beantwortet werden müssen. Dabei werden Themen aus der Makropathologie, der Histopathologie, der Molekularpathologie, der Zytopathologie, der Infektionspathologie/Mikrobiologie, der Obduktionspathologie sowie der Qualitätssicherung aus organisatorischer Sicht abgeprüft.

Zum Bestehen der Prüfung müssen 60 % der Gesamtpunktzahl erreicht werden, wobei in der Histologie, Zytologie und Infektionspathologie/Mikrobiologie mindestens 50 % der möglichen Punkte erreicht werden müssen. Viele Auszubildende absolvieren die Facharztausbildung bereits im vorletzten Ausbildungsjahr. So haben sie bei einem Fehlversuch innerhalb des Facharztausbildungszeitraumes noch eine erneute Möglichkeit, an der Prüfung teilzunehmen.

## Facharztausbildung in Deutschland

Nach erfolgreich beendetem Medizinstudium kann nach Erwerb der Approbation direkt mit der Facharztausbildung begonnen werden (Abb. 1).

Diese gliedert sich in eine 24-monatige Basisweiterbildung, in der grundlegende Kompetenzen des Faches erworben werden sollen.

Daran schließt sich die 48-monatige Weiterbildung zum Facharzt an. Hier soll das Wissen vertieft und weitere Fachkompetenzen erworben werden. Dabei werden die erforderlichen Fallzahlen über die gesamte Ausbildungsdauer berechnet. So muss man im Rahmen der Ausbildung in Deutschland 150 Obduktionen, 15.000 histologische Untersuchungen, darunter auch molekularpathologische Untersuchungen, 500 Schnellschnittuntersuchungen sowie 10.000 zytologische Untersuchungen, davon 5000 aus der gynäkologischen Exfoliativzytologie vorweisen können. Am Ende der Facharztausbildung und nach erreichen der Fallzahlen kann man sich zur Facharztprüfung anmelden, die von den jeweiligen Ärztekammern der Bundesländer durchgeführt wird. Es handelt sich zumeist um eine mündlich-praktische Prüfung. Die Inhalte werden individuell von den Prüfern bestimmt und beinhalten in der Regel Fallbeispiele aus der Makropathologie, der Zytopathologie und der Histologie sowie grundlegende Fragen zu den rechtlichen Grundlagen des Faches, beispielsweise zum Obduktionsrecht.

Neben der Struktur der Facharztausbildungen gibt es aber noch weitere Unterschiede, die für die Standortwahl entscheidend sein können. Diese werden in den folgenden Abschnitten betrachtet.

## Wissenschaftliche Möglichkeiten

Studierende in Österreich erwerben aufgrund des Diplomstudiums mit dem Abschluss lediglich ein Berufsdoktorat. Dieses entspricht in der grundsätzlichen Wertigkeit nicht dem Doktorgrad, der im Rahmen einer medizinischen Promotion in Deutschland erworben wird. Daher ist die Verbreitung von PhD-Programmen für den Erwerb eines PhD-Titels deutlich stärker ausgeprägt als in Deutschland. Im Gegenzug sind in Österreich „Clinician Scientist Programme“, also eine strukturierte wissenschaftliche Arbeit neben der Assistenzarztausbildung, weniger stark verbreitet [1]. Es muss auch bedacht werden, dass die Gesamtmenge der verfügbaren Fördermittel in Österreich geringer ist als in Deutschland [5] und Programme für translationale Forschungsvorhaben, die auch Themenbereiche der Pathologie betreffen können, in Österreich weniger stark entwickelt sind [8].

Dies hat zur Konsequenz, dass Forschende in Österreich teils einem höheren Publikationsdruck und einem höheren Druck bei der Einwerbung von Drittmitteln ausgesetzt sind [4]. In Österreich sind WissenschaftlerInnen oftmals stärker an den Standort gebunden und insgesamt weniger mobil [2], was in Verbindung mit den in der Regel kleineren Universitäten die Auswahl geeigneter Bewerber für wissenschaftliche Stellen bei eigenen Projektvorhaben stärker beschränkt. Die verminderte Mobilität führt insgesamt auch zu einer etwas geringeren internationalen wissenschaftlichen Vernetzung von Standorten in Österreich [3], was sich möglicherweise auf eine wissenschaftliche Karriere auswirken kann.

## Vergleich der Facharztausbildung in Deutschland und Österreich

Beim Vergleich der Facharztausbildungen sind verschiedene Aspekte zu beachten. Zunächst sind die grundlegenden Eintrittsvoraussetzungen verschieden, da in Österreich vor Beginn der eigentlichen Facharztausbildung die Basisausbildung absolviert werden muss. Daneben ist die Facharztausbildung in Österreich durch den mehrteiligen modularen Aufbau stärker strukturiert und reguliert. Dies zeigt sich beispielsweise bei der genauer definierten Reihenfolge der zu absolvierenden Ausbildungsabschnitte und den definierten Fallzahlen, die eine Bedingung für den Übertritt von der Sonderfach-Grundausbildung in die Sonderfach-Schwerpunktausbildung sind. Außerdem muss beachtet werden, dass in Österreich die Mikrobiologie ein Teil des Facharztes für klinische Pathologie und Molekularpathologie ist. In Österreich sind die Ausbildungsinhalte in der Sonderfach-Schwerpunktausbildung stärker definiert und ermöglichen eine einfachere Schwerpunktsetzung in Fachbereichen, die in Deutschland oftmals nur wenig in der Facharztausbildung abgebildet werden wie Molekularpathologie oder Hämatopathologie. Außerdem kann im Rahmen der Facharztausbildung durch das wissenschaftliche Modul über 9 Monate ein Forschungsprojekt innerhalb der Facharztausbildung umgesetzt werden, was in dieser offiziellen Form in Deutschland nicht besteht. Hier können aber auch teils längere Zeiten in der Forschung für den Facharzt angerechnet werden [7]. Im Gegensatz zu Österreich bestehen in Deutschland keine einheitlichen länderübergreifenden Regelungen für die Anerkennung von Ausbildungsleistungen und die Rahmenbedingungen sind oftmals nicht so klar geregelt wie im wissenschaftlichen Modul der österreichischen Facharztausbildung [7]. Besonders groß ist der Unterschied bei der Facharztprüfung, die in Österreich schon während der Ausbildung absolviert werden kann und aus einer schriftlichen Prüfung besteht, während in Deutschland erst nach Ableisten aller Zeiten und Fallzahlen zur Facharztprüfung angemeldet werden kann, die dann mündlich-praktisch durchgeführt wird. Aufgrund des Ärztemangels in der Pathologie sind nach bestandener Facharztprüfung die Aussichten auf eine Anstellung in beiden Ländern sehr hoch [6]. Neben der reinen fachlichen Ausbildung sind bei einem Wechsel aber auch andere Standortaspekte wie wissenschaftliche Karrieremöglichkeiten zu berücksichtigen.

## Fazit für die Praxis

- Die beiden Systeme unterscheiden sich gerade in Eingangsvoraussetzungen und auch im Grad der Formalisierung.
- Wenn man eine stärker strukturierte Ausbildung bevorzugt, kann eine Ausbildung in Österreich infrage kommen.
- In Österreich ist auch die Mikrobiologie Teil des Facharztes klinische Pathologie und Molekularpathologie.
- Die Facharztprüfung in Österreich ist eine schriftliche Prüfung und kann bereits während der Facharztausbildung absolviert werden.
- Die Entwicklungsmöglichkeiten einer wissenschaftlichen Karriere können in Österreich abhängig vom Standort etwas geringer sein als in Deutschland.
- Die Zukunftsaussichten sind in beiden Ländern aufgrund des Fachkräftemangels im Fach Pathologie ausgezeichnet.
