# Erratum zu: Facharztausbildung in Deutschland und Österreich im Vergleich

**DOI:** 10.1007/s00292-025-01530-x

**Published:** 2026-01-13

**Authors:** Steffen Ormanns, Andrea Brunner-Véber, Michael Günther

**Affiliations:** 1 Institut für Allgemeine Pathologie, Medizinische Universität Innsbruck, Müllerstraße 44, 6020 Innsbruck, Österreich; 2 Tirol Kliniken, Innpath Institut für Pathologie, Anichstraße 35, 6020 Innsbruck, Österreich

**Erratum zu:**

**Pathologie 2025**

10.1007/s00292-025-01504-z

Eine initiale Version dieses Beitrags enthielt abschnittsweise Aussagen, die als pauschalisierend angesehen werden könnten, da sie die tatsächliche Situation möglicherweise nicht vollständig widerspiegeln. Sie gehörten nicht zum Kernthema des Artikels. Um Missverständnisse bei der Interpretation dieser aus den Sozialwissenschaften stammenden Aussagen zu vermeiden, die keinen direkten inhaltlichen Bezug zur Facharztausbildung in Österreich und Deutschland aufweisen, haben sich die Autoren entschlossen diese Abschnitte zu entfernen.

Die einzelnen Änderungen im Überblick:

- 1. Seite, Untertitel

Der Blick über die Grenze eröffnet neue Perspektiven: Kultur, Karrierepfade und Chancen

*wurde geändert in:*

Der Blick über die Grenze eröffnet neue Perspektiven: Karrierepfade und Chancen

- 1. Seite, Zusammenfassung
  - 2. Satz

Für einen Wechsel ist das Wissen um die Unterschiede im Aufbau und den Anforderungen der Facharztausbildung sowie um Differenzen in der Arbeitskultur und den Möglichkeiten für eine wissenschaftliche Karriere von großer Bedeutung.

*wurde geändert in:*

Für einen Wechsel ist das Wissen um die Unterschiede im Aufbau und den Anforderungen der Facharztausbildung sowie den Möglichkeiten für eine wissenschaftliche Karriere von großer Bedeutung.

- 5. Satz

Zudem zeigen sich Unterschiede in der Arbeitskultur sowie geringe Differenzen bei den wissenschaftlichen Entwicklungsmöglichkeiten, die in die Entscheidung einbezogen werden sollten.

*wurde geändert in:*

Zudem zeigen sich geringe Unterschiede in den wissenschaftlichen Entwicklungsmöglichkeiten, die in die Entscheidung einbezogen werden sollten.

- Schlüsselwörter

Interkulturelle Differenzen · Pathologie · Wissenschaft · Berufseinstieg · Facharztprüfung

*wurde geändert in:*

Pathologie · Wissenschaft · Berufseinstieg · Facharztprüfung

- 1. Seite, mittlere Spalte, 2. Absatz

Daher ist die Kenntnis über Unterschiede in den Ausbildungssystemen und mögliche kulturelle Differenzen zwischen den beiden Ländern von großer Bedeutung, da diese starken Einfluss auf die Entwicklungsmöglichkeiten am jeweiligen Standort nehmen können.

*wurde geändert in:*

Daher ist die Kenntnis über Unterschiede in den Ausbildungssystemen zwischen den beiden Ländern von großer Bedeutung, da diese Einfluss auf die Entwicklungsmöglichkeiten am jeweiligen Standort nehmen können.

- 3. Seite, mittlere Spalte, letzter Satz im Absatz „Wissenschaftliche Möglichkeiten“

Die verminderte Mobilität führt insgesamt auch zu einer etwas geringeren internationalen wissenschaftlichen Vernetzung von Standorten in Österreich [3], was besonders für eine wissenschaftliche Karriere hinderlich sein kann.

*wurde geändert in:*

Die verminderte Mobilität führt insgesamt auch zu einer etwas geringeren internationalen wissenschaftlichen Vernetzung von Standorten in Österreich [3], was sich möglicherweise auf eine wissenschaftliche Karriere auswirken kann.

- 3. Seite, mittlere Spalte, Absatz „Kulturelle Unterschiede“
  - Kulturelle Unterschiede

Bei einem Wechsel zwischen beiden Ländern sollten auch kulturelle und sprachliche Unterschiede mit in die Entscheidung einbezogen werden, da diese die Zusammenarbeit mit den vor Ort tätigen KollegInnen und dem technischen Personal beeinflussen können. In weiten Teilen Österreichs ist es beispielsweise üblich, auch im beruflichen Umfeld Dialekt zu sprechen, was eine Verständigung anfangs erschweren kann[5]. Des Weiteren kann man gerade im Umgang mit gleichgeordneten und teils auch untergeordneten KollegInnen in Österreich auch unterschwelligen Antipathien gegenüber Deutschen ausgesetzt sein[5]. Akademischen Titeln wird in Österreich traditionell eine viel größere Bedeutung zugeschrieben, da der mit ihnen verbundenen hierarchischen Abgrenzung ein größeres Gewicht beigemessen wird [4]. Beachtet werden muss ebenso eine weniger konsensorientierte Führungskultur, bei der Diskussionen eher als Formalität und weniger der Lösungsfindung dienen[4]. Hier steht dann im Gegensatz zur deutschen Kultur weniger die Suche nach einem gemeinsamen Konsens im Fokus, sondern vielmehr lediglich das formale Durchführen einer Diskussion [4]. So kann es vorkommen, dass die anschließende Entscheidung dann vollkommen unabhängig vom Ausgang der vorangegangenen Besprechung getroffen wird [4]. Dies liegt daran, dass bei der finalen Entscheidung die Kompetenz der beteiligten Personen und objektive Kriterien oftmals eine weniger große Rolle spielen als deren hierarchische Position [4]. Als Reaktion darauf erfolgt dann bei einem Dissens kein weiterer Diskurs, sondern lediglich ein passiver Boykott der Beschlüsse durch diejenigen, die mit der Entscheidung nicht einverstanden sind [4]. Dieser teils deutlich autoritärer anmutende Führungsstil steht in starkem Kontrast zu einer stärkeren Vermischung von privaten und beruflichen Inhalten im Arbeitsalltag [4]. Zudem muss bedacht werden, dass in Österreich deutlich weniger direkt kommuniziert wird und gerade Ablehnung von Vorschlägen selten klar formuliert wird [6]. Vielmehr wird bei einem Dissens auf teils inhaltlich fragwürdige Argumente zurückgegriffen, anstatt diesen offen anzusprechen [6]. Daneben sollte bedacht werden, dass Pünktlichkeit in Österreich weniger streng gelebt wird als in Deutschland [4]. Die genannten Unterschiede können je nach Region unterschiedlich stark ausgeprägt sein. Gerade in der Kommunikation sollte man sich jedoch dieser Unterschiede bewusst sein, um unangenehme Überraschungen zu vermeiden [5, 6].

*Dieser Absatz wurde gelöscht.*

- 4. Seite, linke Spalte, letzter Satz im Absatz „Vergleich der Facharztausbildung in Deutschland und Österreich“

Neben der reinen fachlichen Ausbildung sind bei einem Wechsel aber auch andere Standortaspekte wie wissenschaftliche Karrieremöglichkeiten und kulturelle Unterschiede zu berücksichtigen.

wurde geändert in:

Neben der reinen fachlichen Ausbildung sind bei einem Wechsel aber auch andere Standortaspekte wie wissenschaftliche Karrieremöglichkeiten zu berücksichtigen.

- 4. Seite, mittlere Spalte „Fazit für die Praxis“, Punkt 6

Bei einem langfristig angelegten Wechsel müssen besonders kulturelle Unterschiede beachtet werden, die großen Einfuss auf die Zusammenarbeit am Standort und somit auch auf die weitere berufliche Entwicklung nehmen können.

*Dieser Punkt wurde gelöscht.*

- 4. Seite, Untertitel (Englisch)

Cross-border perspectives: culture, career paths, and opportunities

*wurde geändert in:*

Cross-border perspectives: career paths, and opportunities

- 4. Seite, Zusammenfassung (Englisch)
  - 2. Satz

When considering a career change, it is important to be aware of the differences in the structure and requirements of specialty training programs as well as the differences in work culture and scientific career opportunities.

*wurde geändert in:*

When considering a career change, it is important to be aware of the differences in the structure and requirements of specialty training programs and in scientific career opportunities.

- 5. Satz

In addition, before making a final decision, one must take into account the cultural differences inherent to the workplace and the disparities in scientific development prospects.

*wurde geändert in:*

In addition, before making a final decision, one must take into account the disparities in scientific development prospects.

- Keywords

Intercultural differences · Pathology · Science · Career entry · Specialist examination

*wurde geändert in:*

Pathology · Science · Career entry · Specialist examination

Durch die Änderung dieser Textstellen mussten auch die Referenzen neu sortiert werden:

- Seite 5, linke Spalte
  - [4] Dunkel A, Meierewert S (2004) Culture standards and their impact on teamwork—An empiricalanalysis of Austrian, German, Hungarian and Spanish culture differences. J East Eur Manag Stud:147–174
  - [5] GrethJ, Köllen T (2016) Perceived Anti-Germanism in Austria. Stud Ethn Natl 16:40–62
  - [6] Grzega J (2008) A Few Notes on Conversational Patterns in Germany and Austria. J Eurolinguistix 5:13–22

Diese Referenzen wurden gelöscht.

*Die folgenden Referenzen wurden um nummeriert:*

- Referenz 7 wurde geändert in Referenz 4
- Referenz 8 wurde geändert in Referenz 5
- Referenz 9 wurde geändert in Referenz 6
- Referenz 10 wurde geändert in Referenz 7
- Referenz 11 wurde geändert in Referenz 8

Der Originalbeitrag wurde korrigiert.

